# Disease screening profiles and colostrum management practices on 16 Irish suckler beef farms

**DOI:** 10.1186/s13620-014-0029-7

**Published:** 2015-01-16

**Authors:** James O’Shaughnessy, Bernadette Earley, Damien Barrett, Michael L Doherty, Paul Crosson, Theo de Waal, John F Mee

**Affiliations:** Animal and Bioscience Research Department, Animal & Grassland Research and Innovation Centre, Teagasc, Grange, Dunsany, Co. Meath, Ireland; School of Veterinary Medicine, University College Dublin, Belfield, Dublin 4, Ireland; Animal and Bioscience Research Department, Animal & Grassland Research and Innovation Centre, Teagasc, Moorepark, Fermoy, Co. Cork Ireland; DAFM, Sligo Regional Veterinary Laboratory, Doonally, Co. Sligo, Ireland; Livestock Systems Department, Animal & Grassland Research and Innovation Centre, Teagasc, Grange, Dunsany, Co. Meath, Ireland

**Keywords:** Beef, BVD, Colostrum, Herd health, Liver fluke, Rumen fluke, Suckler

## Abstract

**Background:**

Calf output is a key element in determining the profitability of a suckler beef enterprise. Infectious agents such as Bovine Virus Diarrhoea (BVD) virus, colostrum management and parasitic challenge can all affect calf output. Prior to the national BVD eradication programme, there was little published information on either the prevalence or effect of BVD in Irish beef herds. There is little published information on colostrum management practices in Irish commercial beef herds and there have also been few studies published on the prevalence of liver fluke or rumen fluke infection in Irish beef herds. Sixteen farms participating in the Teagasc/Farmers Journal BETTER farm beef programme were used in this study. Fourteen herds were screened for the presence of BVD virus in 2010 using RT-PCR. In 13 herds, blood samples were collected from calves (2–14 days of age) in November 2011 - April 2012 to determine their passive immune status using the zinc sulphate turbidity (ZST) test, while in 12 herds, blood and faecal samples were taken in order to determine the level of exposure to gastrointestinal and hepatic helminths.

**Results:**

The overall prevalence of BVD virus-positive cattle was 0.98% (range 0 - 3% per herd, range 0.6 - 3.0% per positive herd). Eighteen of the 82 calves (22%) sampled had ZST values less than 20 units (herd mean range 17.0 – 38.5 units) indicating a failure of passive transfer. The overall animal-level (herd-level) prevalence of liver fluke and rumen fluke infection in these herds was 40.5% (100%) and 20.8% (75%), respectively.

**Conclusions:**

The potential costs associated with the presence of animals persistently infected with BVD virus through the increased use of antibiotics; the rate of failure of passive transfer of colostral immunoglobulins and the high prevalence of liver fluke infection in these herds highlight that some Irish suckler beef farms may not be realizing their economic potential due to a range of herd health issues. The use of farm-specific herd health plans should be further encouraged on Irish suckler beef farms.

## Background

A key component of profitable suckler beef production is calf output. In Ireland, a target of 0.95 calves weaned per female mated is the desired production goal [1]. Thus, any factors affecting calf output either directly through increased calf mortality, reduced calf performance or indirectly through reduced herd reproductive performance will affect both farm profitability and sustainability.

Previously, an expert opinion Delphi study and a farmer priority identification survey were undertaken to determine diseases/conditions of importance to Irish beef and dairy farmers [2]. Bovine Virus Diarrhoea (BVD) (ranked first), diseases of young calves (ranked second) and parasitic conditions (ranked fourth) were all categorised as diseases/conditions of concern to beef farmers in the farmer priority identification survey.

Infectious agents such as BVD virus [3,4], poor colostrum management [5,6] and parasitic challenge due to gastrointestinal and hepatic helminths [7-9] can all potentially affect calf output.

Prior to the commencement of the national BVD eradication programme (http://www.animalhealthireland.ie/page.php?id=3), there was only limited information on the prevalence of BVD virus in the Irish cattle population [10]. More recently, the annual losses due to BVD infections in the Irish suckler beef herd were estimated to be €29.1 million [11].

With regard to colostral management in suckler beef calves, previous Irish studies have mainly been conducted in one research centre [12-15] where the effects of dam breed, diet and method of colostrum administration on subsequent calf serum immunoglobulin concentration were investigated. However, one study reported on the quality of colostrum of suckler beef cows and on calf serum immunoglobulin concentrations on 19 commercial farms and reported that 29% of calves had failure of passive transfer of colostral immunoglobulins (FPT) [16].

The prevalence of gastrointestinal and hepatic helminth infections in Irish suckler beef herds is largely unknown with only national disease surveillance reports, which do not differentiate between dairy and beef herds [17], currently available. In a study of 98 culled cows sent to one Irish abattoir [18], it was reported that 56% of cows had liver fluke detected on post mortem examination, with an additional 9% of cows’ livers indicating pathology due to liver fluke infection. Fifty per cent of cows were recorded as having faecal egg counts (FEC) less than 50 eggs per gram (epg) (50 epg being the limit of detection of the method used).

In the case of rumen fluke, the prevalence of infection in Irish cattle appears to be on the increase [19,20]. However, we have limited information on the prevalence of infection in Irish beef herds [1,20]. Faecal samples were analysed for the presence of paramphistome eggs at regular intervals over a 15 month period in one beef herd in the south east of the country [20]. The peak prevalence of infection (57%) was recorded during the summer months with the prevalence declining over the autumn period. Despite the high prevalence of infection during the summer months, no clinical signs of disease were recorded in cattle.

The objective of this study was to determine the following; 1) the animal- and herd-level prevalence of BVD virus-positive cattle and any potential changes in expenditure on antibiotics due to the presence of persistently infected (PI) animals on a farm, 2) to document the colostrum management practices and their effects on the incidence of adequate passive transfer in calves and 3) the prevalence of gastrointestinal and hepatic helminth infections in cattle on farms participating in the BETTER farm beef technology programme (http://www.teagasc.ie/advisory/better_farms/beef/) [1].

## Methods

### Study herds

A brief description of the programme is provided below.

Participants were enrolled in the BETTER farm programme in September 2008. Farmers invited to join the programme were identified based on three main selection criteria: 1) farm location (a wide national geographic spread was required), 2) a willingness to adopt new farm management practices and 3) a willingness to engage in dissemination activities. A further consideration was that the farms would be representative of the suckler farming population in terms of financial performance.

### Farm details

The farms were located in the south east (n = 4), mid west (n = 3), midlands (n = 3), north west (n = 2), north east (n = 2) and south west (n = 2). In 2010 the mean (s.d.) herd size, stocking rate and farm size of these study farms was 68 cows (27.6), 2.0 livestock units/hectare (ha) (0.3) and 64.3 (21.6) adjusted ha, respectively. Cows of predominantly Limousin, Charolais, Simmental and Belgian Blue genotypes accounted for 50%, 21%, 17% and 5% respectively, of all recorded dam breeds on these farms during the period 2009–2010. Charolais, Limousin and Belgian Blue were the breeds of sire for 35%, 29% and 26%, respectively; of all recorded calves born on these farms over the same two year period. Fourteen study farms had both spring (February - April) and autumn/early winter (August - November) calving herds, with the two remaining herds being solely spring and autumn/early winter calving herds, respectively.

### BVD virus screening

The programme management team recommended that participating farmers screen their herds for BVD virus. Fourteen of the 16 participants agreed to screen their herds in 2010 (February-December) for the presence of BVD virus using RT-PCR technology performed on ear biopsies (Enfer Labs, Naas, Co. Kildare, Ireland) with two farmers declining to participate. On ten farms whole-herd screens were performed, while on the other four farms one of the following categories were screened; 1) all breeding stock, 2) replacement heifers and calves, 3) calves only and 4) spring-calving cows and their calves only. Veterinary financial account information (details of farm visits and expenditure on medicines) was sought (with the permission of the farmer) from the veterinary surgeon of the farmer with the greatest number of BVD virus-positive cattle, all of which were later confirmed as PI. This account information was analysed for expenditure on antibiotics and the number of visits to the farm undertaken by the veterinary surgeon to treat clinically ill calves during the period when PI calves were present on the farm. This period was compared to the same period 12 months later when PI cattle were not present on the farm.

### Colostrum management

All 16 study farmers were contacted in September 2011 to ascertain their willingness to participate in a study into colostrum feeding practices and neonatal immunity. Thirteen farmers agreed to participate in the study. The three farmers that did not participate in the study did so for the following reasons; 1) farmer had left the programme, 2) farmer had no calves available for sampling as his herd was an early autumn calving herd and 3) farmer did not perceive any benefit in sampling calves. Calves on the 13 farms were sampled between November 2nd 2011 and April 16th 2012. A convenience sample of calves between two and 14 days of age were blood sampled on the day of the visit. Farms were only visited when as many calves as possible were available for sampling on the day of the visit. In some cases, due to the staggered nature of the calving season on some farms, farms had to be visited a second time so that a sufficient number of calves could be sampled. The aim was to sample six calves per farm. Blood was collected via direct jugular venipuncture and samples were transported to the laboratory and stored at −20°C, until analysis in the Sligo Regional Veterinary Laboratory. The zinc sulphate turbidity test (ZST) was performed at 20°C with the turbidity subsequently measured at 520 nm using a spectrophotometer [21]. Samples were also analysed for total protein and gamma glutamyl transferase (GGT**)**. Although calf serum total protein < 52 g/L and GGT values < 50 U/L are both indicators of FPT [22,23], a ZST value < 20 units was used in this study to define FPT [23].

The method of colostrum administration was also recorded. The following details of each sampled calf were retrieved from the Irish Cattle Breeding Federation (ICBF) database (http://www.icbf.com/): 1) age, 2) sex, 3) sire breed, 4) dam breed and 5) calving ease score [24]. Calving ease scores range from one to four and are recorded by farmers to categorise births into one of four categories: 1 = no assistance, 2 = some assistance, 3 = considerable assistance and 4 = veterinary assistance [25].

### Parasite screening

All 16 farmers were invited to screen their female breeding stock for liver fluke, rumen fluke and gastrointestinal nematodes in the winter of 2011/2012. As three farmers had already treated their female breeding stock with anthelmintics they were excluded while one farmer declined the invitation to sample. On the 12 participating farms, the local veterinary practitioner collected rectal faecal samples and a heparinised blood sample from breeding females between November 14th 2011 and January 9th 2012. Samples were only collected from pregnant heifers and cows that had not received an anthelmintic treatment in the previous six months.

Samples were sent to the Parasitology Section at the Central Veterinary Research Laboratory, Backweston, Celbridge, Co. Kildare, Ireland. Faecal samples were analysed for the presence of fluke eggs (liver and rumen) using a sedimentation technique while FEC were determined using the McMaster technique with a limit of detection of 50 epg. The blood samples were sent to Teagasc, Grange to determine plasma levels of GGT, aspartate aminotransferase (AST) and glutamate dehydrogenase (GLDH). Plasma GGT levels were determined using the International Federation of Clinical Chemistry and Laboratory Medicine (IFCC) method using a kit and protocol supplied by Beckman Coulter (Cat no OSR6120). Plasma AST levels were also determined using the IFCC method using kit and protocol supplied by Beckman Coulter (Cat no OSR6109) while GLDH levels were determined using the Deutsche Gesellschaft für Klinische Chemie method using kit and protocol supplied by Randox Laboratories, U.K., (Cat GL441). All enzymes analyses were performed on an Olympus Analyser AU400 (Beckman Coulter (Irl.) Ltd.). The normal reference ranges for each enzyme are as follows; AST (78–132 U/L), GGT (6.1 - 17.4 U/L) and GLDH (<31 U/L) [26].

#### Statistical analysis

Descriptive statistics were used to quantify the various variables. Using the PROC REG procedure of SAS 9.3, the coefficient of determination (r2) was used to determine the relationship between measures of passive transfer (calf serum total protein, ZST and GGT values) and also the relationship between calf age and ZST.

## Results

### BVD virus

A total of 26 out of 2,652 cattle (0.98%) screened for the presence of BVD virus were found to be virus-positive, with virus-positive animals being identified in 7 out of the 14 farms screened (50%) (range 1–10 per positive herd). The overall prevalence of BVD virus-positive cattle ranged from 0 - 3% per herd and from 0.6 - 3.0% per positive herd. The mean age (range) of BVD virus-positive cattle was 7 months of age (3 weeks - 18 months of age), with 3 cattle < 1 month of age, 12 cattle between 1 and 6 months of age and 11 cattle > 6 months of age. Fifteen of the 26 cattle initially identified as BVD virus-positive cattle were re-tested and all were confirmed as PI. Five of the seven farmers with BVD virus-positive cattle had been vaccinating their cattle against BVD prior to screening in 2010.

The potential economic cost due to BVD infection as judged by changes in antibiotic expenditure was estimated on the farm where the greatest numbers of BVD virus-positive cattle were identified. Ten BVD virus-positive cattle were identified on this farm after screening 432 cattle (herd prevalence 2.3%). This herd had started vaccinating pre-breeding against BVD in 2008. The BVD virus-positive calves were born from September 21st – November 15th 2009 (representing 8% of the August 2009 - April 2010 calf crop). Calves were diagnosed as BVD virus-positive in February 2010. Upon identification as BVD virus-positive, all ten animals were isolated in a shed on the farm and were subsequently retested and confirmed as PI. One of these PI animals died due to pneumonia in March 2010. The remaining nine animals (mean age nine months of age) were slaughtered on July 6th 2010, realizing an average slaughter price of €254 each. This figure represented approximately 40% of the market value of non-diseased stock of similar age at that time [27]. There was a 34% decrease in expenditure on antibiotics on calves during the corresponding period twelve months later, when no PI animals were present on the farm, despite an increase of 40% in the number of calvings in that period due to increased cow numbers (Table 1). The total expenditure on antibiotics on all livestock on this farm was reduced by 47%.

**Table 1:** **Veterinary farm visits to sick calves, antibiotic spend and number of calvings on a farm with ten cattle persistently infected (PI) with BVD virus**

	**21st Sept. 2009- 6** ^**th**^ July 2010 (A*)	21st Sept. 2010- 6th July 2011 (B**)	^**1**^ **Percentage increase/decrease**
Calvings (number)	82	115	40
Vet. calls to sick calves (number)	12	10	−17
Total antibiotic spend (€)	1302	696	−47
Antibiotic spend on calves (€)	972	637	−34

^1^The percentage increase/decrease between the period when PI cattle were born and were present on the farm until slaughter and the corresponding period twelve months later. ((A-B) ÷ A) × 100. *Period of time that ten PI cattle were born and present on the farm until slaughter. **Corresponding period twelve months later.

### Colostrum management

Seventeen farm visits were undertaken and 82 blood samples were collected. Six calves on average per farm were blood sampled (range 4–8 calves). Fifty six calves were recorded as having suckled their dams unassisted, 24 calves were administered fresh colostrum after calving (nipple-bottle feeding or by use of an oro-esophageal tube), one calf received frozen colostrum, while the method of colostrum administration for another calf was unrecorded. Eighteen of the 82 calves (22%) sampled had ZST values less than 20 units while herd mean ZST values ranged from 17 to 38.5 units (Table 2), with only five farms having all calves that were sampled having ZST values greater than 20 units. Of the 82 calves sampled, 27 calves (33%) had serum total protein values less than 52 g/L while 13 calves (16%) had GGT values less than 50 units, respectively. Of the calves that suckled unassisted or were administered colostrum, 11 (20%) and six (25%) calves, respectively, had serum ZST values less than 20 units. The mean (s.d.) calving ease score for calves who suckled unassisted and those that were administered colostrum was 1.2 (0.4) and 1.6 (0.8), respectively. The r2 values for each set of variables were as follows; 1) ZST and calf serum total protein (r2 = 0.65, P < 0.001), 2) GGT and ZST (r2 = 0.21, P < 0.001), 3) GGT and serum total protein (r2 = 0.24, P < 0.001) and 4) calf age and ZST values (r2 = 0.10, P = 0.289).

**Table 2:** **Calf serum total protein, gamma glutamyl transferase (GGT) and zinc sulphate turbidity (ZST) values in suckler beef calves sampled on 13 Irish suckler beef farms**

**Farmer ID**	**No. calves sampled**	Calf age at sampling* (days)	Total Protein* ^**1**^ **(g/L)**	GGT* ^**2**^ **(U/L)**	ZST* ^**3**^ **(units)**
1	7	7.1 (3.4)	52.9 (7.5)	284.7 (238.4)	20.1 (13.1)
2	8	6 (3.3)	52.5 (7.2)	161.4 (115.1)	26.1 (10.0)
3	8	6.2 (2.6)	61.3 (5.6)	229.1 (137.7)	36.7 (6.3)
4	6	7.0 (2.2)	51.7 (7.6)	175.7 (264.0)	17.0 (11.2)
5	6	4.3 (2.0)	57.1 (5.6)	216.2 (163.3)	33.8 (5.9)
6	5	10.2 (4.2)	62.3 (10.1)	147.6 (102.7)	34.2 (13.2)
7	7	9.3 (3.0)	52.7 (11.8)	140.1 (94.7)	19.7 (11.8)
8	5	11.6 (1.7)	61.9 (7.1)	179.0 (148.7)	34.8 (6.3)
9	5	11.0 (2.3)	57.5 (8.1)	77.4 (60.3)	29.4 (11.5)
10	4	3.3 (0.5)	68.4 (11.3)	539.8 (292.3)	38.5 (10.4)
11	7	5.7 (3.0)	62.0 (11.7)	267.6 (299.6)	29.1 (11.4)
12	6	9.5 (4.2)	55.2 (4.3)	266.8 (219.8)	28.8 (5.7)
13	8	6.5 (4.1)	51.2 (5.0)	156.5 (69.8)	26.5 (7.6)
Overall	82	7.4 (3.6)	56.8 (9.0)	211.6 (193.5)	28.3 (11.2)

*Denotes herd mean (s.d.).

^1^Individual calf serum total protein < 52 g/L is an indicator of failure of passive transfer of colostral immunoglobulins (FPT) [22]. ^2^Individual calf GGT values < 50 U/L and ^3^ZST values < 20 units also indicate FPT [23]. 4In this study, an individual calf ZST value was used to define FPT.

### Parasite screening

A total of 169 cattle were faecal sampled (range 9–20 per farm) on 12 farms. Of the cattle where parity was recorded (n = 157), faecal samples were taken from the following four parity groups: 1) in-calf beef heifers (parity 0) (n = 35), 2) 1st calvers (parity 1) (n = 29), 3) 2nd calvers (parity 2) (n = 33) and 4) 3rd calvers or older (parity 3 or above) (n = 60). Liver fluke and rumen fluke eggs were detected in the faeces of 40.5% (range 15.5 - 68.8% per herd) and 20.8% (range 0.0 - 50.0% per herd, range 6.3 - 50% per positive herd), respectively, of all sampled cattle (Table 3). The overall mean herd FEC was 9 epg (range 0–22 epg per herd; range 3–22 epg per positive herd) with 85% of cows sampled having strongyle egg counts less than 50 epg. The overall mean animal (herd mean range) AST, GGT and GLDH plasma levels were 87 (67–101), 25 (14–40) and 14 (6–23) U/L, respectively. Sixty three per cent of cows sampled had GGT values elevated above the normal range, with 62% of cows that had liver fluke eggs detected in their faeces also having elevated GGT values.

**Table 3:** Hepatic enzymes*, strongyle egg counts and prevalence of liver and rumen fluke infection in suckler beef cows and heifers on 12 Irish suckler beef farms

**Herd ID**	**Sampling month**	**Location**	**No. cows and heifers sampled**	AST** ^**1**^ **(U/L)**	GGT** ^**2**^ **(U/L)**	GLDH** ^**3**^ **(U/L)**	Mean strongyle egg count**	**No. animals liver fluke + ve per farm (%)** ^**4**^	**No. animals rumen fluke + ve per farm (%)** ^**4**^
1	November	South-west	20	101 (17.4)	31 (40.9)	23 (30.5)	18 (29.4)	6 (30.0)	10 (50.0)
2	November	South-east	16	100 (25.2)	30 (33.2)	15 (14.3)	9 (20.2)	11 (68.8)	5 (31.3)
3	November	North-east	13	71 (25.4)	23 (5.1)	9 (5.9)	0 (0.0)	2 (15.4)	3 (23.1)
4	November	North-east	12	80 (17.6)	28 (14.0)	9 (6.6)	17 (32.6)	4 (33.3)	1 (8.3)
5	November	West	16	98 (28.5)	40 (37.0)	20 (16.3)	3 (12.5)	5 (31.3)	3 (18.8)
6	November	North-west	16	96 (21.7)	26 (21.9)	16 (12.1)	7 (25.8)	6 (37.5)	1 (6.3)
7	November	North-west	12	-	-	-	4 (14.4)	8 (66.7)	6 (50.0)
8	December	South-east	16	80 (23.2)	20 (7.0)	11 (8.7)	3 (12.5)	4 (25.0)	0 (0.0)
9	December	South-east	16	79 (10.4)	14 (7.6)	11 (6.3)	6 (17.1)	11 (68.8)	5 (31.3)
10	December	South-east	11	93 (34.6)	22 (10.0)	13 (16.0)	5 (15.1)	5 (45.5)	1 (9.1)
11	December	Midlands	12	67 (8.4)	14 (5.9)	6 (3.0)	13 (31.1)	4 (33.3)	0 (0.0)
12	January	Midlands	9	81 (13.0)	20 (6.7)	9 (3.5)	22 (36.3)	2 (22.2)	0 (0.0)
Overall	November-January	All farms	169	-	-	-	9 (22.6)	68 (40.2)	35 (20.7)

- Values not available. *aspartate aminotransferase (AST), gamma glutamyl transferase (GGT) and glutamate dehydrogenase (GLDH), **values expressed as arithmetic mean (s.d.). ^1^AST normal range (78–132 U/L), ^2^GGT normal range (6.1-17.4 U/L) and ^3^GLDH normal range (<31 U/L) [26]. ^4^Individual faecal samples were examined for fluke eggs using a sedimentation technique and based on their morphological characteristics were classified as either liver or rumen fluke eggs.

## Discussion

In studies of this nature with a small sample size, it is difficult to generate associations between study findings and the wider suckler beef farming population. Although farms in this study were representative of the national suckler herd in term of financial performance, these study herds were considerably larger than the average suckler beef farm in Ireland where mean herd size is typically 14 cows [28]. Nonetheless, irrespective of the representativeness of the study herds, this study highlights a number of important findings with respect to suckler herd management in Ireland.

The high prevalence of BVD virus-positive cattle (0.98%) in these Irish suckler beef herds, which was determined prior to the initiation of the national BVD eradication programme (Statutory Instrument No. 118/2014) was a surprising finding and contrasts with the prevalence in the first year of the national BVD eradication programme (0.77%, http://www.animalhealthireland.ie/page.php?id=3).

In a previous study on these farms [1], we reported that, on average, 70 cattle were purchased per farm over a two year period (2009–2010). This was a high level of purchasing given that the mean herd size in 2010 was 68 cows. On three of the seven farms where BVD virus-positive cattle were identified during screening, purchased cattle were either BVD virus-positive themselves or gave birth to calves that were subsequently identified as BVD virus-positive. This occurred despite the fact that the majority of farmers (5/7) with BVD virus-positive cattle had been vaccinating their livestock against BVD prior to screening. This again highlights the risks associated with purchased livestock [29] and that vaccination alone may not be sufficient to prevent the infection of livestock and the generation of PI animals from occurring [30].

Previously, the costs of BVD in Irish suckler beef herds were estimated using bio-economic modelling [11]. Losses were estimated to range from €10 to €38 per cow/year depending on vaccination status and herd size. One of the costs considered in the model was veterinary costs. In this study, we compared a period when ten PI animals were present on a farm and the corresponding period 12 months later when these animals had been removed. Despite a 40% increase in the number of calvings in the period when no PI cattle were present on the farm, there was an overall reduction of 47% in expenditure on antibiotics. This is surprising as it would be expected that with increased livestock numbers, morbidity might potentially increase due to higher infection challenge in sheds or at pasture. Although disease patterns on farms can change from year to year, being dependent on many factors, the large decline in antibiotic spend (47%) coincided with removal of PI cattle. In addition, the poor performance of the nine PI calves that survived until slaughter further highlights the direct economic loss associated with retaining these animals for any length of time on a farm.

Based on the results of the ZST test, 22% of calves had FPT. The rate of FPT in this study was similar to another Irish study of 19 commercial suckler beef farms where 29% of calves that were allowed to suckle their dams unassisted had FPT [16]. In contrast to the previous study [16], the calving score ease was recorded for each calf in the present study as dystocia can have an important influence on subsequent calf serum immunoglobulin concentration. This is due to the respiratory acidosis generated in a calf as a result of dystocia which may affect their ability to absorb immunoglobulins [31] while reduced calf vigor can delay the onset of suckling. Eleven (20%) of the 56 calves that suckled their dams unassisted had FPT despite a low mean calving score (1.2). These results indicate that suckler beef calves that experience minimal dystocia and are allowed to suckle unassisted are at risk of FPT. Similarly, a Canadian study which included only unassisted births [32], reported that 22% of calves that suckled their dams without assistance had FPT. Therefore, assisting suckler calves to suckle colostrum appears to be favourable practice in ensuring these calves receive adequate colostrum [13,15]. In both these studies, it was reported that suckler beef calves achieved high rates of passive transfer of immunoglobulins provided they were assisted to suckle or fed a volume of colostrum based on their live weight (40–50 ml/kg live weight) using a stomach tube within one hour post-partum. It is difficult to explain the rate of FPT in calves that were administered colostrum after calving; six (25%) of the 24 calves administered colostrum had FPT. However, four of these calves experienced assistance in delivery (mean calving score of 2.25) which may have subsequently influenced colostrum absorption while the other two calves were marginally below the cut-off of 20 ZST units (16 and 18 ZST units).

The animal-level prevalence of liver fluke infection in this study was 40.5% (range 15.5 - 68.8% per herd). This was lower than what has previously been reported under Irish conditions [18]. Based either on the direct recovery of liver fluke or identifying the pathology associated with liver fluke infection on post mortem examination, an overall prevalence of infection of 65% was reported in culled cows sent to one Irish abattoir [18]. However, only 37% of cows’ livers in the autumn period (October - December) in that study, which was similar to our sampling time period (November - January), had liver fluke identified on post mortem examination. Although detecting liver fluke eggs in faecal samples is highly specific in diagnosing liver fluke infection [33], it may lack the necessary sensitivity to diagnose infection; sensitivity ranges from 66 - 69% [33,34]. Therefore, it is possible that the prevalence of liver fluke infection in these herds was underestimated.

The plasma activities of three enzymes (GGT, GLDH and AST) were also measured at the time of faecal sampling for fluke eggs. Although none of these enzymes are specific indicators of fasciolosis *per se*, they do provide information on the extent of the pathology to both the liver parenchyma and the biliary system. Both AST and GLDH indicate damage to the liver parenchyma, while GGT, which indicates damage to the biliary system, has been shown to increase approximately eight weeks after initial challenge with liver fluke [35] and plasma levels may subsequently remain elevated for many months post-challenge [36]. Only plasma GGT values in this study were elevated above the normal range. This may have been the result of pathology induced by adult liver flukes.

The animal-level prevalence of rumen fluke in these herds was 20.8% (range 0% to 50% per herd). The range in herd prevalence for rumen fluke infection in these herds, which was similar to liver fluke, probably reflects differences in local grazing conditions. Similar to liver fluke, rumen fluke also requires a snail as an intermediate host to complete its lifecycle. Formerly*, Paramphistomum cervi* was suspected to be the main rumen fluke species affecting cattle and sheep in Ireland [37]. However, recent studies have indicated that *Calicophoron daubneyi* is the main species affecting ruminants in Ireland [20,38,39]. As this species of rumen fluke shares the same intermediate host as liver fluke, similar pasture control measures can be adopted for both species. Although reports indicate that the prevalence of rumen fluke infections in Irish cattle are increasing [19,20], the clinical significance of rumen fluke infection in Irish cattle has yet to be determined.

An interesting finding in the present study was the higher prevalence of liver fluke infection compared to rumen fluke infection. This is in contrast to national surveillance reports in cattle [17,40] where the prevalence of rumen fluke infections was considerably greater than liver fluke infections as judged by the identification of fluke eggs in faecal samples submitted for examination. In both reports, the prevalence of liver fluke infection was approximately seven per cent whereas the prevalence of rumen fluke infection ranged from 35 - 39%. The findings of the present study may indicate a more targeted approach to the treatment of rumen fluke infections as opposed to liver fluke infection on these farms. This however, was not investigated. The FEC recorded in this study are in accordance with previous studies conducted in suckler beef herds where dams had negligible strongyle egg counts [41-43].

## Conclusions

Prior to this study, there was little information on the potential effect of BVD virus infections on calf morbidity in Irish suckler beef herds. There was also little information on colostral management practices, or on the prevalence of infection with common gastrointestinal and hepatic parasites.

Approximately 20% of calves that were born experiencing minimal dystocia and who subsequently suckled their dams unassisted had FPT. Therefore, it is advisable that all suckler beef calves are encouraged to suckle their dams after birth irrespective of the nature of the delivery.

The experience of one farmer in this study where calf morbidity increased and expenditure on antibiotics increased considerably in association with the presence of a number of BVD virus-positive animals on a farm, which were later confirmed as PI, highlights the financial impact that may result from the presence of PI animals on farms.

Farmers participating in the parasite study all had patent liver fluke infections identified in their herds with the majority also having evidence of rumen fluke infection. Suitable and sustainable control strategies specific to farms need to be implemented to control these parasites more effectively on Irish suckler beef farms.
